# Ethical implications of developing RNA-based therapies for cardiovascular disorders

**DOI:** 10.3389/fbioe.2024.1370403

**Published:** 2024-03-15

**Authors:** Mihaela Hostiuc, Alexandru Scafa, Bogdan Iancu, Daniela Iancu, Oana-Maria Isailă, Oana Mihaela Ion, Ana Stroe, Camelia Diaconu, Dragos Epistatu, Sorin Hostiuc

**Affiliations:** ^1^ Carol Davila University of Medicine and Pharmacy, Bucharest, Romania; ^2^ Open Sci World, Bucharest, Romania; ^3^ Wessex Clinical Genetics Service, Southampton, United Kingdom; ^4^ University College London, London, United Kingdom

**Keywords:** RNA-based therapies, cardiovascular disorders, autonomy, cost-to-benefit analysis, bioethics

## Abstract

The awareness concerning RNA-based therapies was boosted significantly after the successful development of COVID-19 vaccines. However, they can potentially lead to significant advances in other areas of medicine, such as oncology or chronic diseases. In recent years, there has been an exponential increase in the number of RNA-based therapies that were evaluated as potential treatments for cardiovascular disorders. One of the areas that was not explicitly assessed about these therapies is represented by their overall ethical framework. Some studies evaluate ethical issues of RNA-based treatments in general or targeting specific disorders (especially neurodegenerative) or interventions for developing RNA-based vaccines. Much less information is available regarding the ethical issues associated with developing these therapeutic strategies for cardiovascular disorders, which is the main aim of this study. We will focus our analysis on three main topics: risk-benefit analysis (including the management of public awareness about these technologies), and justice (in both research and clinical medicine).

## 1 Introduction

Nucleic acid-based therapies are a class of therapies using exogenous DNA or RNA molecules designed to produce a therapeutic effect. Although RNA therapies have gained significant attention recently, mainly due to their association with COVID-19 vaccines (Roncati and Corsi, 2021), their development has been ongoing for several decades. The first proof-of-concept experiments were conducted 3 decades ago using messenger RNA (mRNA) to express a protein *in vivo* artificially. The initial work used an intramuscular injection to deliver one of three mRNAs transcribed *in vitro* to mice (Wolff et al., 1990). Protein expression from injected mRNAs was found to be equally efficient using DNA-encoded vectors (based on protein levels expressed from injected nucleic acid). The following pivotal study used laboratory-made vasopressin mRNA to temporarily correct a rat model of diabetes insipidus (Jirikowski et al., 1992). From experimental works with mRNA, we now have a diverse range of biomolecules known as “RNA therapeutics.” They are based, on the use of ribonucleic acids to modify the expression or activity of their target molecules and, through this, to modulate various biological pathways involved in treating multiple disorders (Kim, 2022). Their target may be at the pre-mRNA level (using U1snRNA vectors or splicing modulation/correction by using antisense oligonucleotides (ASOs)), at the mRNA level (gene inhibition through siRNAs, microRNAs, or ASOs), or the DNA level (CRISPR-Cas9) (Coutinho et al., 2019).

One of the areas was not explicitly evaluated concerning these therapies was their ethical framework. Some studies assessed these issues either in general, by targeting specific disorders (especially neurodegenerative) or the development of vaccines (Sherley et al., 2020; Rhodes, 2021; Al-Sheboul et al., 2022). Much less information is available regarding the ethical issues associated with developing these therapeutic strategies for cardiovascular disorders, which is the main aim of this study. Their importance resides from the very high prevalence of cardiovascular disorders, which are known to affect in a larger proportion high-income country, having increased available financial resources allocated to healthcare, allowing a potential inclusion in clinical guidelines of these highly expensive therapies. These therapies also have specific ethical issues, are summarized in Table 1. We will focus our analysis on risk-to-benefit analysis and justice, as we considered them to be the most relevant at this stage of development.

**TABLE 1:** Ethical issues associated with RNA-based therapies.

General principle	Details
Beneficence and non-maleficence	Uncertainties related to beneficence due to potentially undesirable side-effects (Ebbesen et al., 2008; Ansah, 2022)
	Proper evaluation of the risk/benefit ratio (Ebbesen et al., 2008)
	Potential maleficence for the placebo group (Alqahtani et al., 2021)
Autonomy	Respect of autonomy in clinical trials (Ebbesen et al., 2008)
	Proper information/understanding check (Hostiuc, 2022)
Justice	Just inclusion and exclusion criteria in clinical trials
	Just coverage through healthcare insurance (Rüger et al., 2020)
	Changing current paradigms of care (King and Bishop, 2017)
	Lack of international harmonization (Cuende et al., 2022)
Trust	Increased/decreased public trust (King and Bishop, 2017)
Compassion	Compassionate use of experimental drugs (Mendoza et al., 2016)
	Compassionate use or expanded access (Paumgartten and Oliveira, 2020)

## 2 Ethical issues

### 2.1 Risk-benefit analysis

The benefits of any therapeutic intervention should be evaluated in relation with potential risks. Any intervention has costs risks, and burdens. The risks may be evaluated semiquantitatively, depending on parameters like severity, imminence of harm, or probability. Potential benefits may be assessed semiquantitatively, using probability, magnitude of benefit, or beneficiary (Beauchamp and Childress, 2001; Hostiuc, 2022). Afterward, a risk/benefit ratio may be constructed and used to justify a specific intervention. There is no universally applicable risk-benefit ratio, as it also depends on disease severity. If there is no cure and the prognosis is lethal without intervention, a riskier approach may be allowed from an ethical standpoint, as is generates a benefit not obtainable through any other means. Specifically, RNA-based therapies are still associated with long-term uncertainties about their potential benefits and harms. In theory, these risks and benefits are extensively evaluated through clinical trials before a drug is approved. However, they cannot establish a definite risk-benefit profile before being clinically approved. A risk leading to increased concerns, especially for the public, is mutagenesis. A known example is represented by the X-linked severe combined immunodeficiency, caused by the integration of a delivered RNA virus in the host’s genome, as a provirus, causing increased oncogenesis. (Hacein-Bey-Abina et al., 2003). This risk has been considered minimal for RNA-based therapies in cardiovascular disorders (Magadum et al., 2019), but there are still uncertainties in the long term (Ebbesen et al., 2008). To further decrease/eliminate this risk were proposed the use of lentivirus-instead of gamma retrovirus vectors (Schenkwein et al., 2020), viral hybrid-vectors (plasmid replicon pEPI, EBV-based replicons, Semliki Forest Virus replicons) (Zhang et al., 2014) or non-viral vectors. Another potential risk is the complexity of targeting the molecule to a specific tissue, which may cause unforeseen effects, from minor to lethal (Kratzer et al., 2022), due to cross-reactions and potential toxicity in other tissues/organs. Mipomersen use was associated with an increased rate of side effects, such as severe injection site injuries or hepatotoxicity, when evaluated in patients with hypercholesterolemia and atherosclerosis on a lipid-lowering therapy associated with apheresis (Waldmann et al., 2017). For the same drug, Reeskamp et al. found that 21% of subjects developed transaminitis compared to 1% in the placebo group, suggesting a significant risk of hepatotoxicity (Reeskamp et al., 2019). The risk for hepatotoxicity is generated because most genetic targets for cardiovascular disorders currently used by RNA therapies are in the liver. Both siRNA and ASOs have been engineered to bind asialoglycoprotein receptors expressed on the surface of hepatocytes. This has the advantage of lowering the drug concentration and, subsequently, adverse reactions in other organs but increases liver-related risks (Arsenault, 2022).

The benefits should be evaluated in relation to the best alternative therapy and the lethality of the disease. A new, RNA-based treatment for a disorder with an efficient treatment should not be considered for a first-line treatment due to its long-term uncertainties. If the pathology is lethal due to the absence of efficient treatment, or if the standard treatment is associated with significant burdens and adverse reactions, RNA-based therapies may be seen as having a more favorable risk-benefit ratio and, therefore, will be morally justified to prescribe/recommend them. Another possible factor that increases the risk acceptance of a drug is its compassionate use, namely, its administration to treat a life-threatening condition for which there is no authorized drug available when some data suggest that it might be helpful (Goyal et al., 2020).

Hype and over-optimism regarding novel technologies were shown to overestimate the potential benefits and underestimate the potential harms of novel drugs. There are numerous contributors to the hype surrounding novel treatments - media coverage through targeted campaigns sponsored by pharmaceutical companies, the preference of journals to publish positive results, a lack of motivation for researchers (who are usually funded) to disclose all information regarding adverse effects, the statistical cover-up of negative information through carefully drafted results, or the use of acronyms suggesting a potential benefit (King and Bishop, 2017). For example, the study aimed to evaluate Eplontersen for transthyretin-mediated amyloid cardiomyopathy, which has the abbreviation CARDIO-TTRANSform (CARDIO-TTRansform 2023: A Study to Evaluate the Efficacy and Safety of Eplontersen (Formerly Known as ION-682884, IONIS-TTR-LRx and AKCEA-TTR-LRx) in Participants With Transthyretin-Mediated Amyloid Cardiomyopathy (ATTR CM), n.d.), implying a transformative therapy. Another study, about the effects of Inclisiran for atherosclerotic cardiovascular disease associated with increased LDL-C despite receiving maximally tolerated statin therapy is known as VICTORION-INITIATE (A Randomized 2023 Study to Evaluate the Effect of an “Inclisiran First” Implementation Strategy Compared to Usual Care in Patients With Atherosclerotic Cardiovascular Disease and Elevated LDL-C Despite Receiving Maximally Tolerated Statin Therapy (VICTORION-INITIATE) (V-INITIATE), n.d.), suggesting its “victory” upon other treatments. In practice, in numerous clinical trials using RNA technologies, methodological issues mainly focused on efficacy, safety, unclear hypotheses, or even scientific validity were found (Paumgartten and Oliveira, 2020; Bejar et al., 2022).

Sometimes the public openly opposes these therapies, underestimating the clear benefits of this approach to new drug/vaccine development. Fieselmann *et al.* for example, found the main reasoning for opposing a COVID-19 vaccine were low perceived benefits, low subjective risk of acquiring the disease, health concerns (potential side effects, lack of long-term studies, getting cancer, infertility, death, genetic changes/damages), information deficits (the available information was considered scarce and incomprehensible), systems mistrust (authorities, public stakeholders, representatives of the pharmaceutical industry - including physicians), and spiritual and religious beliefs (Fieselmann et al., 2022). Holford *et al.* found the following psychological constructs to be associated with anti-vaccination argument endorsement: conspiracy mentality, general district, pseudoscientific beliefs, centrality of religion, moral absolutism, trait fear, prosocial behavioral intention, alternative epistemology, general reactance, free market ideology, traditionalism, populism, and perceived vaccination risk (Holford et al., 2023). To this general reluctance must be added the high treatment, which could easily augment the resistance of the general population against them, as it may be seen as a method to reallocate significant healthcare funds to highly controversial therapies, for which common drugs are already available. For RNA-based therapies, the underestimation of the benefit by the general population was not extensively evaluated, most likely because of the novelty of the approach and the related costs.

### 2.2 Justice

Justice can be seen as equity in the distribution of healthcare-related resources in a society. There are many theories of justice in healthcare, each with specific advantages and disadvantages, depending on the unique features of a particular issue. The most well-known and discussed approach was attributed to Aristotle, according to which we should treat the equals equally and the unequal - unequally (Broadie and Rowe, 2002). This is known as the formal principle of justice, as it does not specify who the equals should be treated equally, not the criteria based upon which two individuals are not equal. A more recent attitude, which has gathered significant support in healthcare is the Rawlsian approach. Rawls argues that the principles of justice should be constructed under a veil of ignorance, under which we would know nothing about ourselves. By not knowing anything about who we are, what diseases we have, or our financial status, we would try to develop a system of values to generate minimal discomfort, irrespective of the potential state we would be in at a particular time. The fundamental principles we would build under this veil of ignorance would be, according to Rawls, the principle of the greatest equal liberty (each person has an equal right to the most extended base liberty compatible with the liberty of others) and the principle of difference (social and economic inequalities should satisfy two primary conditions - to be attached to open offices and position under a fair equality of opportunity and to generate the most significant benefit for the least advantaged citizens) (Rawls, 2009). However, These approaches are normative - they tell us what should be done to act justly but not how which is essential to apply the principle (Hostiuc, 2022). Carr argues that the principle of justice has two elements: a material one, which ensures the presence of a justificatory condition to establish the just character of a treatment, and a formal one, which generalizes the requirement for all similar, relevant cases (Carr, 1981). For this analysis, justice should be evaluated in relation to research and clinical practice.

In research, the principle is respected by establishing just inclusion and exclusion criteria (Ebbesen et al., 2008), providing optimal availability of health services to study participants, including vulnerable populations in trials while respecting their need for additional safeguards that are dependent on the type of vulnerability, or providing post-trial access to the developed drugs if they are found to be better compared to the previous best therapy for that particular disorder (Wibawa, 2021), by proving unlicensed drugs to single patients to fulfill special/unique needs (Synofzik et al., 2022), if no other treatments are potentially helpful. Suppose the studies are conducted in countries with limited resources. In that case, there is an increased risk of exploitation of vulnerable subjects, an issue that should be adequately evaluated and managed before the beginning of the study. Even if, for the study’s validity, it should be multicenter [72], a careful selection of the countries/regions where the study will gather its participants should be seen as an initial step to validate the study protocol. It should be noted that these therapies are costly for now, and their availability in countries with more modest financial resources will be minimal. Therefore, simply conducting the study in these countries, especially if therapeutic alternatives are available or if the efficacy potential is not evaluated before the study as significant, should be discouraged, as it may be seen as primarily exploitative (Denny and Grady, 2007; Chennells, 2015; Hostiuc, 2022). Based on the principle of community-based equipoise, the only exception would be to extend beneficial treatments to subjects unable to benefit from them in the clinical phase (Emanuel et al., 2008). By analyzing the locations of most studies regarding RNA-based strategies (using the search engine from clinicaltrials.gov), we saw that most were conducted in high-income countries, with significantly fewer locations in middle-income regions. However, by analyzing the inclusion and exclusion criteria, we identified some that could be considered less than optimal from an ethical point of view. For example, for Volanerosen, one of the exclusion criteria was to “Have any other conditions in the opinion of the investigator which could interfere with the participant participating in or completing the study” (COMPASS, 2023). Even if this may be justifiable from a medical point of view, as numerous conditions may significantly alter the effects of a particular drug on a specific subject, a comprehensive exclusion might decrease the representativeness of the topics for the general population. In another trial, this time for Mipomersen (A Study of the Safety and Efficacy of Two Different Regimens of Mipomersen in Patients With Familial Hypercholesterolemia and Inadequately Controlled Low-Density Lipoprotein Cholesterol (FOCUS FH)), a similar exclusion criterion was much better handled, by specifying the health problems that should be excluded, and by limiting them to the recent past “significant health problems in the recent past including heart attack, stroke, coronary syndrome, unstable angina, heart failure, significant arrhythmia, hypertension, blood disorders, liver disease, cancer, digestive disorders, Type I diabetes, or uncontrolled Type II diabetes” (Mipomersen, 2023). Another example of perfectly justifiable exclusion criteria with the same aim is for ARC1779. This RNA aptamer was evaluated for the treatment of patients with von Willebrand factor-related platelet disorders, which states: “Any major, active health problem, e.g., cancer or heart disease, which could render the patient medically unstable during the period of participation in the study” (ARC1779, 2023).

Gene-based therapies are known to be very expensive, with overall costs in the six to seven figures. For example, Hemgenix costs 3.5 million USD, Zolgensma 2.1 million, or Kymriah - 475.000 USD (Haseltine, 2023). RNA-based therapies approved for cardiovascular disorders are apparently cheaper by comparison. For example, Mipomersen has a price of around 7.000$, Volanesorsen - around 14.000E, and Inotersen - around 8.000$. However, these are the prices for one dose, the annual costs being extremely prohibitive. For example, the yearly cost of Inotersen treatment is around 420.000 USD (Executive Summary, 2020). From a cost-utility analysis, the pricing is not justifiable. For the same drug, it was found that a price decrease of at least 88% would be needed for it to be cost-effective by using a 50.000$ per QALY threshold (Executive Summary, 2020). When recommending an expensive treatment, we should consider not only the costs of the drug *per se* but also ancillary costs (transportation to a facility having that resource, the time needed to be spent to receive it, time lost to various administrative procedures, *etc.*) (King and Bishop, 2017). Due to these extremely high costs, the main parameters based on which justice can be evaluated is the financial one. Other potentially relevant issues, such as cultural differences in addressability, or racial, ethnic and religious acceptance, might gather traction once the costs would decrease to make them potentially useful for larger groups of patients.

An efficient RNA-based therapy may be directed toward a common or a rare cardiovascular disease, and it may replace another efficient treatment or represent the only efficient treatment. Depending on the combination of these two parameters and the overall costs, significant allocations of resources toward these techniques may be considered as respecting or not the principle of justice. If the therapy targets common disorder that has good pharmacological alternatives, the inclusion of these treatments as a standard option would significantly increase the costs without obvious clinical benefits over the standard treatment (no significant decrease of DALYs/increase in QALY), and therefore an allocation of healthcare resources toward them will not respect the principle of justice. Suppose the therapy is directed toward a common disorder whose treatment does not have an excellent pharmacological approach. In that case, the allocation may be seen as just, but only if it would decrease DALYs in that population or increase QALY. In this instance, the costs of the RNA-based drug should be reduced by increasing the production scale, which would also benefit other medications based on the same strategy. If the therapy is directed toward a rare disease with good pharmacological alternatives, there is no ethical justification for its inclusion in guidelines as standard treatments. It may, however, be offered as a paid alternative for those patients. This approach would respect their autonomy, as they will be free to choose the best treatment. It is to be noted that some RNA-based therapies have distinct advantages, such as a very long interval between injections and a decreased risk of adverse reactions, making them potentially indicated from a purely clinical point of view for a specific category of patients. It would also encourage pharmaceutical companies to develop further these treatments, which would, in time, optimize production, decrease research times, decrease costs, or lead to the development and commercialization of new drugs. If the therapy is directed toward rare diseases, and there is no pharmacological treatment available, the principle of Rawlsian justice is respected by providing the treatment, irrespective of changes in DALY/QALY, as these types of diseases are usually neglected, and beneficial interventions are rarely developed. Health insurance systems should use a cost-benefit analysis before extending coverage for these drugs, which should consider the consequences of the affected disorder, its prevalence, and alternative treatment options.

## 3 Conclusion

RNA-based therapies for cardiovascular disorders are still a very new topic. However, considering the exponential increase in interest in the last few years and the many molecules being actively developed, we can expect many more to appear in clinical guidelines for various disorders in the coming years. Their widespread use should be accompanied by a proper ethical analysis of their use and by careful dissemination of relevant information to the public to avoid the mistrust that was shown to be associated with COVID-19 vaccines.
